# In Honour of Francis Joseph

**Published:** 1898-12-24

**Authors:** 


### IN HONOUR OF FRANCIS JOSEPH.

This is the Jubilee of the accession of the Emperor of Austria. Except our own Queen there is no other European monarch who is so loved and honoured by his subjects; Germans, Czechs, and Magyars, at war with each other on every other point, unite in personal devotion to the Emperor, who has made, and keeps them while he lives, one great nation, with a place among the great powers of Europe, instead of a trio of small and discordant ones. Of the sorrows of the Emperor one hardly dares to speak. Our Queen, with whom we have compared him, and who alone has occupied her throne longer than he, has had her share of natural griefs. She has lost a beloved husband, and children whose lives were an honour to their high position; but in each case death came naturally, and she could bow to the Divine will without a murmur. But of the bereavements of Francis Joseph who shall speak? Is not this year of Jubilee, when his people are rejoicing that Providence has so long spared to them a monarch whose sole concern is their welfare, marked by the deepest trial of his much-tried life? The assassination of the Empress of Austria roused the sympathy of even those who have no great belief in "the divinity that doth hedge a king." Is it strange that the subjects of the Emperor are desirous that their memorials of his long reign should be of such a nature—religious and charitable—as will be most acceptable to his chastened and unselfish soul? Such a project is now being set up in our midst. In the interchange of travel many Austrians are now coming to our shores. Some of these are rich, others find in Britain an outlet for their special talents; but others, venturing here with the object of bettering their fortune, find themselves occasionally stranded without the means of improving their condition, or even of returning to their home. For the benefit chiefly of these it is proposed to establish in England a "Francis Joseph Institute" in commemoration of the Jubilee of the accession of "His Imperial and Royal Apostolic Majesty the Emperor of Austria and King of Hungary." This institute is really the name of a society, which shall have its chief offices in London, but may from time to time establish branches in such places in the United Kingdom as may be deemed necessary. The society proposes to absorb any smaller Austrian and Hungarian benevolent societies already existing, the increased economy and efficiency to be obtained by such centralising of effort being quite obvious. To mark the fact that the association represents the whole of the dual monarchy, it is laid down in the regulations that the chairman shall be an Austrian and a Hungarian alternately, the appointment being an annual one. The other members of the society shall be life members, who have given a donation of not less than £25 to its funds; annual members, who subscribe annually not less than £1 1s. if they reside permanently in the United Kingdom; and, if not permanently located there, subscribe at least 10s. 6d. There will also be honorary members, not liable for any subscription, such being persons who, in the unanimous opinion of the general board, are deserving of such a compliment in consideration of services rendered in promotion of the objects of the institute.

These objects are all of a philanthropic nature. The society may, if it is deemed advisable, buy or lease premises suitable for an institute, or buy or lease land and build suitable premises upon it. In these premises it may establish a hospital, dispensary, dormitory, or other similar institutions for the relief of the needy and sick, or it may, if authorised by the general board, use the funds at its disposal to provide gratuitous medical relief or medicine, surgical appliances, and the like, or to obtain for its beneficiaries admission to existing hospitals. Destitute persons belonging to the Austro-Hungarian monarchy may also be housed and fed in the institute, and it is proposed to have a library and reading-room, where books, newspapers, periodicals, and works of reference may be consulted free of charge. Other objects of the institute will be the assistance, by grants of money, clothing, or other necessaries, of poor subjects of the dual monarchy in this country, to help them to obtain employment, or, if that be impossible, to assist them to return to their homes. Assistance will also be given to widows and orphans, and, when necessary, their interest will be looked after. Akin to this last is the intention to provide legal assistance for Austro-Hungarian subjects, in such cases as it is thought desirable. Foreigners are inevitably at a disadvantage in the law courts of strange countries. They may be guilty of some breach of legal formality, which may put them at an undeserved disadvantage, or their ignorance of the language may make it difficult for them to obtain their rights. To have the services of a competent lawyer may often enable any man to assert his rights, and a foreigner needs such assistance, even more than a native. Such are the objects of the Francis Joseph Institute. Similar societies for the benefit of the other nationalities already exist in our midst, and do a most useful work, providing for the poor and unfortunate of these that assistance which is given more kindly by, and accepted more gratefully from, a compatriot's hands than a stranger's. To this last accession to their number we bid hearty welcome, and commend it not merely to natives of the dual monarchy in this country, but to all who have interests, direct or indirect, with the Austro-Hungarian Empire.

